# Quantitative signal properties from standardized MRIs correlate with multiple sclerosis disability

**DOI:** 10.1002/acn3.51354

**Published:** 2021-05-04

**Authors:** Matthew R. Brier, Abraham Z. Snyder, Aaron Tanenbaum, Richard A. Rudick, Elizabeth Fisher, Stephen Jones, Joshua S. Shimony, Anne H. Cross, Tammie L. S. Benzinger, Robert T. Naismith

**Affiliations:** ^1^ Department of Neurology Washington University in St. Louis St. Louis Missouri USA; ^2^ Malinckrodt Institute of Radiology Washington University in St. Louis St. Louis Missouri USA; ^3^ Biogen Cambridge Massachusetts USA; ^4^ Imaging Institute Cleveland Clinic Cleveland Ohio USA

## Abstract

**Objective:**

To enable use of clinical magnetic resonance images (MRIs) to quantify abnormalities in normal appearing (NA) white matter (WM) and gray matter (GM) in multiple sclerosis (MS) and to determine associations with MS‐related disability. Identification of these abnormalities heretofore has required specialized scans not routinely available in clinical practice.

**Methods:**

We developed an analytic technique which normalizes image intensities based on an intensity atlas for quantification of WM and GM abnormalities in standardized MRIs obtained with clinical sequences. Gaussian mixture modeling is applied to summarize image intensity distributions from T1‐weighted and 3D‐FLAIR (T2‐weighted) images from 5010 participants enrolled in a multinational database of MS patients which collected imaging, neuroperformance and disability measures.

**Results:**

Intensity distribution metrics distinguished MS patients from control participants based on normalized non‐lesional signal differences. This analysis revealed non‐lesional differences between relapsing MS versus progressive MS subtypes. Further, the correlation between our non‐lesional measures and disability was approximately three times greater than that between total lesion volume and disability, measured using the patient derived disease steps. Multivariate modeling revealed that measures of extra‐lesional tissue integrity and atrophy contribute uniquely, and approximately equally, to the prediction of MS‐related disability.

**Interpretation:**

These results support the notion that non‐lesional abnormalities correlate more strongly with MS‐related disability than lesion burden and provide new insight into the basis of abnormalities in NA WM. Non‐lesional abnormalities distinguish relapsing from progressive MS but do not distinguish between progressive subtypes suggesting a common progressive pathophysiology. Image intensity parameters and existing biomarkers each independently correlate with MS‐related disability.

## Introduction

Multiple sclerosis (MS) is an inflammatory demyelinating disease^1^ associated with neurodegeneration.^2^ MS‐related disability is prevalent and can be substantial.^3^ The diagnostic hallmark of MS is focal demyelinating lesions^4^ that appear as white matter (WM) hyperintensities on T2‐weighted (or FLAIR) magnetic resonance images (MRIs).^5^, ^6^, ^7^ WM lesions (WMLs) have prognostic value in individual patients.^8^ However, lesion burden correlates poorly with clinical disability.^9^ Non‐lesional abnormalities are also of critical importance in MS, yet are difficult to discern on routine MRIs.^10^

MS leads to histopathological abnormalities and microglial activation in WM that are not evident on routine clinical imaging.^11^ These abnormalities partially predict clinical disability.^12^ Gray matter (GM) pathology in both cortical and subcortical structures often begins early in MS^13^, ^14^ and also partially correlates with disability.^15^ However, neither normal‐appearing WM (NAWM) nor GM pathology is readily detectable on routine clinical imaging. Atrophy, including thalamic atrophy, strongly predicts future disability.^16^ However, atrophy is a downstream manifestation of neurodegeneration that reflects the sum of multiple pathologic processes. Specialized techniques such as magnetization transfer imaging,^17^ diffusion tractography,^18^ and quantitative relaxometry^12^ are useful in the study of MS, but these techniques require lengthy MRI acquisitions and specialized analyses. Consequently, assessing the contribution of non‐lesional pathology to disability has previously been possible only in research applications.

Thus, development of non‐lesional imaging biomarkers is of critical importance for assessing disease severity and improving our understanding of the pathological basis of disability. We developed a novel approach based on multimodal voxel intensity normalization that can be applied to standard clinical MRI datasets. This approach enables measurement of abnormalities in non‐lesional tissue that are theoretically informationally equivalent to quantitative relaxometry.^19^ We evaluated this technique in a large, previously collected dataset of T1‐weighted and T2‐weighted FLAIR images with regard to differentiating individuals with MS from non‐MS controls, distinguishing between relapsing versus progressive MS subtypes, and determining correlations with disability measures. We hypothesized that T1‐weighted and T2‐weighted images, following appropriate transform, would contain quantitative measures of tissue integrity allowing for the identification of abnormal non‐lesional tissue. We tested this hypothesis in a large observational dataset and assessed the extent to which the information provided by our novel analysis methodology is complimentary to conventional biomarkers of MS pathology (i.e., WML burden and atrophy).

## Methods

### Standard protocol and patient enrollment

Data were obtained from the MS PATHS network, comprising 10 sites in the United States and Europe. The MS PATHS database contains clinical and imaging data collected from a large, heterogenous MS population as part of routine patient care. MS PATHS participants agree to share pseudoanonymized data with the research sponsor and the network investigators under the auspices of individual Institutional Review Boards after providing written informed consent. The MS PATHS database includes clinical performance measures and 3D T1‐weighted and 3D FLAIR images (both 1‐mm isotropic voxels) acquired on 3T Siemens scanners using a standardized imaging protocol. After local approval by the Institutional Review Board at Washington University in St. Louis, data used in the present analyses were drawn from Data Cut 6 (Downloaded 12/5/2018).

### MS PATHS inclusion and exclusion criteria

Inclusion criteria for all MS PATHS participants included clinically confirmed MS and ability to provide informed consent. Additional criteria applied in the present analysis are detailed below.

### Clinical characterization in MS PATHS

Clinical measurements closest to the imaging session were used. Many (1206) were performed the same day. In all cases, <180 days separated sessions (mean 43 days, st. dev 50 days).

#### MS subtype

Patients characterized themselves as having one of the following types of MS: relapsing–remitting (RR), secondary progressive (SP), primary progressive (PP), or progressive relapsing (PR). The nomenclature regarding subtypes is based on the original MS PATHS study design. All four subtypes were initially analyzed separately, but statistical analysis revealed that progressive subtypes were indistinguishable. Accordingly, subsequent analyses focused on relapsing remitting versus progressive subtypes.

#### Patient determined disease steps

The patient determined disease steps (PDDS) is a self‐reported disability score with a strong correlation (*r* = 0.78) to the Expanded Disability Status Scale.^20^ Patients rate their disability from 0 to 8, with 0 corresponding to normal, (1) – *mild disability*, (2) – *moderate disability*, (3) – *gait disability*, (4) – *early use of a cane*, (5) – *late use of a cane*, (6) – *bilateral support*, (7) – *wheelchair or scooter dependent*, and (8) – *bedridden*.

#### Objective performance testing

Performance tests were administered during clinical visits on an iPad using the Multiple Sclerosis Performance Test (MSPT).^21^ Measures were designed to simulate components of the multiple sclerosis functional composite and include the processing speed test, a digital version of the symbol digit modalities test^22^; the manual dexterity test, simulating the 9‐hole peg test^23^; and the walking speed test, a timed 25‐foot walk.^24^ Results from the testing session closest to the MRI date were used in the present analysis.

#### Quality of life evaluation

Patients ranked their subjective symptoms using the computer adapted version of the Neuro‐QoL.^25^ This instrument assesses 12 domains of health‐related quality of life such as cognitive function and upper extremity use. Results from the questionnaire closest to the scanning session were used.

### Healthy controls data collection

Healthy control data were collected at MS PATHS institutions as part of an ongoing concurrent substudy. Subjects aged 21–60 were recruited to be age, gender, and race matched to the clinical MS PATHS population. Exclusion criteria included comorbid neurological conditions (e.g., stroke, epilepsy, and Alzheimer's disease), migraine requiring medication, autoimmune disease, pregnancy, or history of human immunodeficiency virus. Healthy control subjects underwent the same data collection procedures (MSPT and MRI) as the MS patients.

### Image processing and analysis

Basic image processing steps are described here; more detail is available in the Supplemental Material. T1 MP‐RAGE and 3D FLAIR (1‐mm^3^ voxels) underwent affine alignment, brain extraction, and bias field correction.^26^

**Figure 1:**
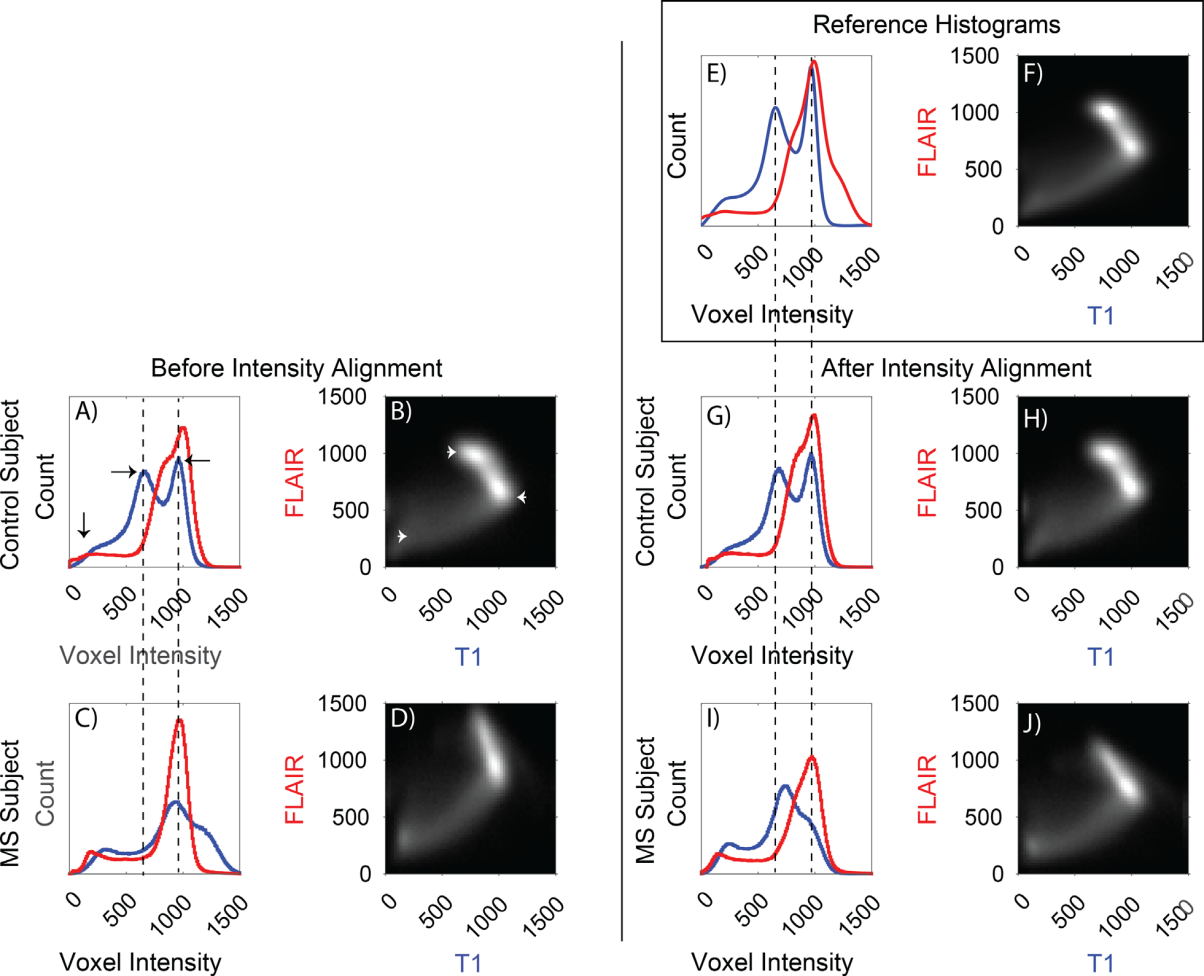
Demonstration of intensity alignment in control and MS patients. (A) Univariate histogram for a randomly selected control participant demonstrates T1w intensity in blue, with highest voxel counts for intensity at approximately 200 (for CSF), 600 (for gray matter), and 1000 (for white matter). Flair intensity in red indicates highest voxel numbers were at approximately 200 (for CSF), 700 (for white matter), and 1100 (for gray matter). (B) The same control, bivariate, voxel‐wise intensity histogram (higher voxel counts are bright) demonstrates a clustering of voxels at T1w intensity of approximately 600 and FLAIR intensity of approximately 1100 (gray matter, left arrowhead), and another clustering of voxels at FLAIR intensity of approximately 700 and T1w intensity of approximately 1000 (white matter, right arrowhead). (C and D) Histograms from a randomly selected RRMS patient demonstrate abnormalities in location and distribution of peaks. Alterations in location and distribution of T1w and FLAIR in the bivariate plot (in D) are not just due to the disease but also due to lack of normalized signal intensities owing to uninteresting scan parameters. In (A and C), the dashed lines indicate the nonalignment of the peak loci in the T1w distribution (blue line). Based upon the normal reference created from 101 healthy controls (E and F), intensity alignment was affine transformed for the control participant (G and H), and the MS patient (I and J). The MS patient marginal histogram now demonstrates better normalization of T1w intensity (blue line) and broadening of the FLAIR peak (red line) which corresponds to T2‐weighted abnormalities in MS.

The fundamental principle underlying the present analyses is standardization of bivariate intensity (T1w × FLAIR) histogram shape to match normative data. This is a statistical approach to analysis of image intensity distributions. Differences in alignment owing to atrophy do not affect this analysis. Normative data from 101 age‐ and sex‐matched controls imaged using identical procedures were generated. Bivariate histograms were created representing T1w/FLAIR voxel intensities on the horizontal/vertical axes, respectively. Histogram peaks occurred at specific loci in intensity space corresponding to distinct tissue classes (GM, WM, CSF; Fig. 1). Individual participant histograms exhibited grossly similar shapes in healthy controls and MS patients but subtly varied in scale and skew across individuals (dashed lines in Fig. 1).

Intensity normalization of all participants (*n* = 5038) was achieved by affine registration of individual bivariate histograms to the reference histogram. This normalization facilitates tissue classification and comparison. Figure 1 illustrates bivariate histograms in two example participants (1 control, 1 MS patient) before and after intensity normalization. Histogram alignment error in each individual was compared to a fixed criterion determined by visual inspection of a representative sample (Figure S1). Individuals with histogram alignment error above this threshold (*n* = 28, 0.6%) were excluded. Histogram normalization parameters obtained in each participant were applied voxelwise to the T1w and FLAIR data, thereby generating images whose bivariate histograms closely approximated the normative reference.

Intensity normalization generated T1w and FLAIR images in which tissue classes (CSF, WM, GM, lesion) were represented as partially overlapping distributions about centroids in a bivariate intensity space. Tissue segmentation was achieved by implementing a procedure similar to k‐means clustering (described in the Supplemental Material) to compute voxelwise tissue class assignments (Figure S2). Each voxel was classified as either GM, WM, CSF, or WML. A conservative methodology was adopted to identify unconfounded NA voxels, with ambiguous or borderline voxels classified as lesion for this analysis. A separate procedure was used to quantify GM and WM volumes (see *Brain atrophy measures* below; “MSPie” and Figure S2 and S3).

**Figure 2:**
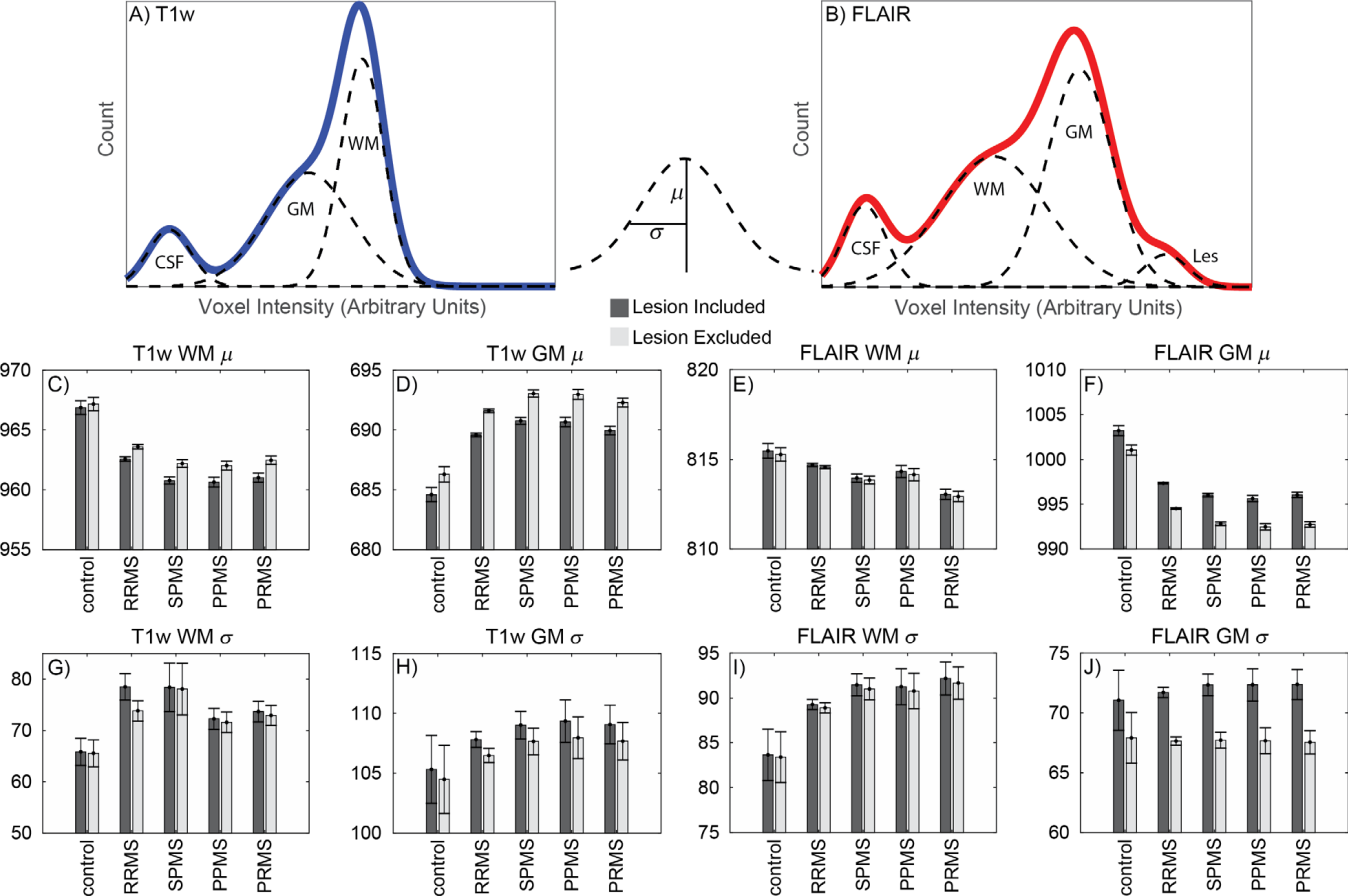
Histogram parameters differentiate MS patients from controls. The top panels (A, B) display simulated data to demonstrate the application of Gaussian mixture models (GMMs) to univariate histograms. Solid blue and red lines correspond to the hypothetically observed T1w and FLAIR intensity histograms, respectively. GMM attempt to model these observed distributions as a sum of Gaussians, these Gaussians are indicated by dashed lines. Each Gaussian is described by a mean (*µ*) and standard deviation (*σ*). These parameters are calculated for each individual participant in WM and GM on the T1w and FLAIR image. The Gaussian corresponding to the CSF is not analyzed. The mean (standard error) of each parameter across participants is shown in the bottom panels (C–J). Dark gray bars correspond to estimates calculated across the entire brain mask; light gray bars correspond to estimates calculated across the brain mask with discrete WM lesions excluded. The principle impact of lesion exclusion on GMM parameters was to reduce within‐tissue class variability (*σ*). As a rule, MS (all subtypes) are associated with increase *σ* in all measures indicating increased signal variability (wider histograms). For *µ* parameters, the direction varies. T1w WM and FLAIR WM and GM appeared darker in MS patients and T1w GM appeared brighter in MS patients.

In principle, statistical measures could be extracted directly from the bivariate histograms. However, MS pathology is commonly described in terms of T1w and FLAIR images. Therefore, to preserve the interpretability of the present findings, we adopted a univariate approach whereby T1 and FLAIR intensity histograms were modeled separately. The normalized data were split into two univariate histograms representing normalized T1w and FLAIR image intensity distributions. These univariate, normalized histograms were subsequently subjected to comparisons of interest, for example, MS patients versus controls. Group comparisons were computed by analysis of normalized, univariate T1w and FLAIR intensity distributions within tissue classes determined by Gaussian mixture modeling (GMM). GMMs applied to T1w and FLAIR intensity distributions provide a signal intensity mean (*µ*) and standard deviation (*σ*) for each tissue class, CSF, GM, WM, and lesion (Fig. 2A and B). These parameters (*µ*, *σ*) from GM and WM derived from the T1w and FLAIR data were analyzed further. GMM fitting was carried out twice, once with lesions included and again with lesions excluded.

### Brain atrophy measures

MS PATHS Image Evaluation (MSPie) automatically segments GM and WM structures and delivers estimates of brain parenchymal fraction (BPF), WM fraction (WMF), GM fraction (GMF), and thalamic volume.^27^ These metrics, included in the MS PATHS repository, were used to estimate atrophy. Of note, lesion masks derived from MSPie and the present lesion score approach are strongly similar (Supplemental Results, Figures S2 and S3).

### Statistical procedures

#### Evaluating differences in mean GMM parameters between Controls and MS patients

The first investigated question was whether GMM parameters differ between MS patients and controls; the second question was whether GMM parameters differ among MS subtypes. ANOVA models were fit with group (control, RRMS, SPMS, PPMS, PRMS) as factor. Each of 8 GMM parameters was fit separately. Thus, the significance level was set as *P* < 0.05/8 = 0.00625. In cases of significant ANOVA models, post hoc pairwise contrasts were extracted to determine between which groups there were significant differences.

#### Evaluating the relationship between biomarkers and disability

We next sought to quantify the relationships between biomarkers (e.g., GMM parameters, lesion volume, and atrophy) and disability as measured by the PDDS. Biomarkers were *Z* scored, thereby facilitating direct comparison of regression *β* values. Each biomarker was separately fit in a linear regression model with intercept and PDDS as modeled factors. The *β* (slope) values were of key interest as they directly reflect the strength of the statistical relationship between biomarkers and PDDS.

#### Identification of unique contributors to disability

The preceding analyses established a relationship between PDDS and biomarkers of interest. A LASSO regression model was fit to isolate variables uniquely contributing to PDDS. LASSO regression eliminates variables contributing redundant information from the model.^28^ To verify the validity of the fit, a hold‐out analysis was performed wherein the model trained on 90% of available data was applied to the previously unseen 10%.

### Data availability statement

MS PATHS data are currently accessible to Biogen or participating healthcare institutions in the MS PATHS program. Analysis code will be made available to qualified researchers upon request.

## Results

### Participant demographics

Complete datasets were acquired in 5038 participants (101 healthy controls and 4937 MS patients). Of these, 5010, including all 101 healthy controls, passed quality control procedures based on alignment of the bivariate intensity histogram. MS patients comprised 68% RRMS, 18% SPMS, 6.5% PPMS, and 8% PRMS groups. Patients with RR subtype were more likely to be younger, female, and less disabled compared to progressive subtypes (Table 1). Rates of disease modifying therapy were similar across subtypes.

**Table 1:** Participant demographics.

	Controls	RRMS	SPMS	PPMS	PRMS
*N*	101	3340	877	321	399
*N* excluded	0	17	8	2	1
Gender, %*F*	75%	75%	73%	64%	64%
Age in years (SD)	41.1 (11.8)	45.2 (11.9)	49.8 (11.1)	50.3 (12.5)	47.0 (12.4)
PDDS (SD)	N/A	1.13 (1.53)	3.54 (1.96)	3.36 (2.29)	2.97 (2.27)
Age at diagnosis (SD)	N/A	34.0 (10.4)	34.8 (11.0)	39.1 (12.3)	33.1 (11.3)
On disease modifying therapy (%)	N/A	76%	69%	77%	67%

Note.

Demographic information for MS PATHS participants and healthy controls. Mean (standard deviation) of selected demographic variables by group and MS subtype. PDDS, patient determined disease steps.

### Gaussian mixture models measure differences between MS patients and controls

Figure 2C–J shows *μ* and *σ* corresponding to GM and WM, in normalized T1w and FLAIR data, in healthy controls, RRMS, SPMS, PPMS, and PRMS. ANOVA found significant MS versus control differences in all but four out of 16 combinations of image type × measure, with or without lesions included (dark or light bars in Fig. 2; Table S1). In particular, T1w mean WM intensity (*μ*) was significantly lower in all MS subtypes (Fig. 2C) compared to controls, indicating diffuse, WM changes in MS. Additionally, T1w WM *σ* was larger (wider distribution) in all MS subtypes compared to controls (Fig. 2G), indicating increased heterogeneity of T1w WM signal. Similarly, FLAIR WM *σ* was wider in all MS subtypes compared to controls (Fig. 2I). These outcomes were the same whether or not lesions were included in the WM measure, indicating that NAWM is abnormally heterogenous in MS. In the case of controls, differences owing to the inclusion or exclusion of lesions are due to the presences of nonspecific WM hyperintensities classified as lesions likely owing to small vessel disease or a similar process. The FLAIR GM *μ* parameter performed best for discriminating control versus MS (Fig. 2F).

**Figure 3:**
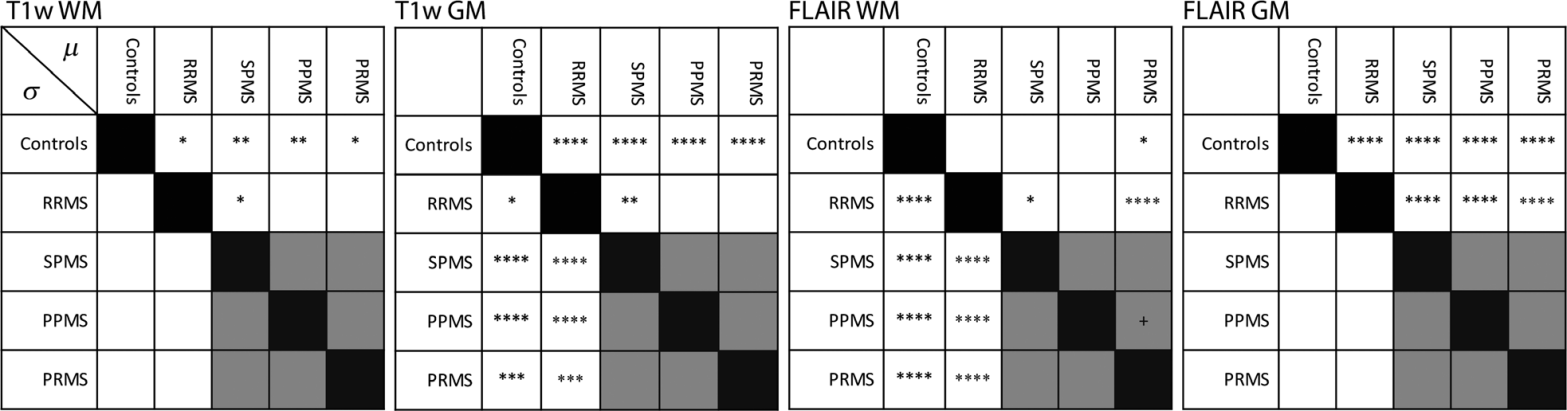
Parametric differences separate MS from controls and relapsing MS from progressive MS. Matrices show the results of post hoc contrasts extracted from the models shown in Table 3. Pairwise comparisons were made between each of the five groups (controls, RRMS, SPMS, PPMS, PRMS) for each parameter. Contrasts relating to the *µ* parameter are shown above the diagonal and contrasts relating to the *σ* parameter are shown below the diagonal. The logarithmic magnitude of the *P* value for the corresponding contrast is represented by number of “*” with ^+^ *P* < 0.05; *P < 10^−2^; **P < 10^−3^; ***P < 10^−4^; ****P < 10^−5^. Empty boxes indicate nonsignificant contrasts. The most significant differences were between controls and all MS subtypes (first row or column) and between RRMS and progressive MS subtypes (second row or column), whereas differences among the three progressive subtypes were unapparent.

Figure 3 shows results obtained by post‐hoc extraction of comparisons from the ANOVA model, with lesions excluded; *μ* and *σ* comparisons are listed above and below the diagonal, respectively. The most significant differences were between healthy controls and all MS subtypes (first row or column of Fig. 3); the next most significant were between RRMS and progressive MS subtypes (second row or column of Fig. 3). Almost no significant differences were found between the three progressive MS subtypes (shaded area of each matrix). Thus, GMM analysis of normalized intensity distributions distinguished patients with MS from healthy controls and RR MS from progressive MS but did not distinguish among progressive MS subtypes.

Differences in mean values are difficult to generalize to the individual subject. We investigated the sensitivity and specificity of the GMM parameters for determining two comparisons: (1) control versus MS and (2) RRMS versus progressive subtypes. Comparisons within progressive subtypes were excluded on the basis of no mean differences being found in the above analysis (Fig. 3). For each GMM parameter and comparison, we calculate the ROC AUC parameter, sensitivity, and specificity (Table 2). Overall, the GMM parameters with the most significant differences in mean value also had the most discriminative value as measured by the ROC. As regards discriminating controls versus MS patients, FLAIR WM *σ* had the largest AUC (0.84). The AUC of FLAIR WM *µ* was only 0.55 indicating that this parameter was minimally discriminative of MS versus controls. The biological interpretation of GMM parameters with near‐chance AUC is uncertain.

**Table 2:** GMM parameter ROC AUC.

	T1 WM *µ*	T1 GM *µ*	T1 WM *σ*	T1 GM *σ*	FL WM *µ*	FL GM *µ*	FL WM *σ*	FL GM *σ*
Control versus MS								
AUC	0.77	0.72	0.66	0.65	0.55	0.82	0.84	0.54
Sensitivity	0,45	0.56	0.68	0.40	0.90	0.76	0.77	0.28
Specificity	0.83	0.77	0.60	0.82	0.13	0.77	0.72	0.82
RRMS versus progressive subtypes								
AUC	0.58	0.55	0.61	0.58	0.53	0.63	0.69	0.51
Sensitivity	0.65	0.31	0.63	0.52	0.66	0.72	0.55	0.74
Specificity	0.49	0.77	0.52	0.62	0.42	0.49	0.66	0.28

Note.

ROC AUC, sensitivity, and specificity for the discrimination of controls versus MS patients and RRMS versus progressive subtypes based on the eight GMM parameters. GMM, Gaussian mixture modeling; MS, multiple sclerosis.

### Relations between lesion‐burden and atrophy biomarkers and disability

**Figure 4:**
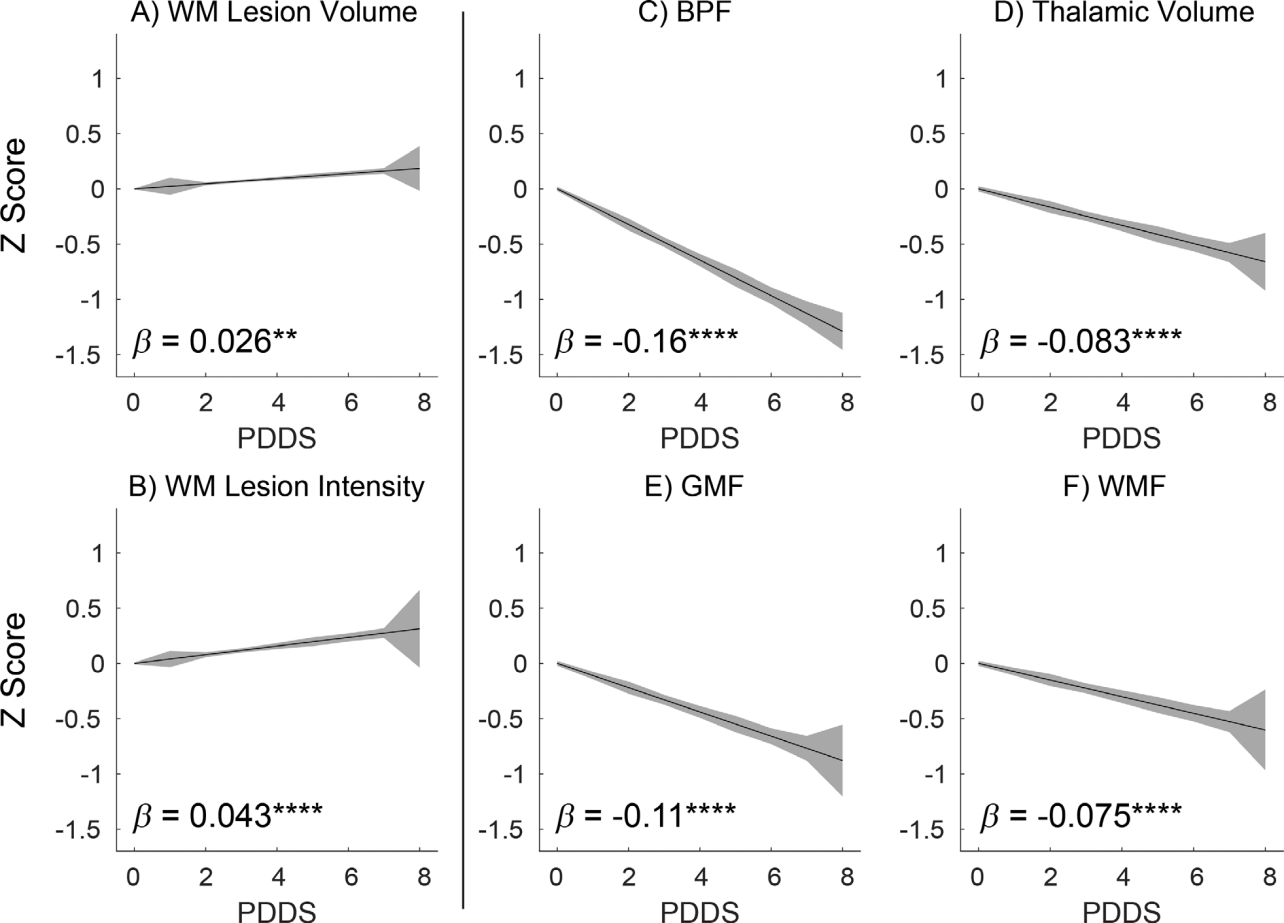
Relationship between established MS imaging biomarkers and MS disability. The relationship between imaging measures of MS patients and the patient‐determined disease steps (PDDS) score are shown graphically, along with linear regression *β* values (shaded areas correspond to 1 SD around the mean predicted value). Imaging measures were *Z* transformed such that different metrics are on the same scale, making *β* values comparable across measures. The first column (A, B) shows the relationship with two measures of WM lesion burden. WM lesion volume is the sum of voxels identified as lesions. WM lesion intensity is WM lesion volume weighted by the lesion intensity (${\tilde{u}}_{l}^{v}=1/n_{L}\sum_{v\in L}u_{l}^{v},$ where *u_l_* is the lesion score and *L* is the set of voxels within a lesion). The remaining panels (C–F) show the relationship between PDDS and brain parenchymal fraction (BPF), thalamic volume, WM fraction (WMF), and GM fraction (GMF). Of note, in all cases the lesion based measures are outperformed by the brain volume based measures. Asterisks indicate *P* values. *P < 10^−^^2^; **P < 10^−^^3^; ***P < 10^−^^4^; ****P < 10^−^^5^.

Figure 4 relates existing lesion‐based biomarkers and brain volume‐based biomarkers to PDDS evaluated over all MS subtypes. The *Z* score (across the entire cohort) of each biomarker was entered into regression analysis with PDDS as the independent variable. The obtained regression coefficients (*β*) quantitate magnitude of the imaging biomarker relation to disability and allow comparison between alternative biomarkers. Among the six evaluated imaging biomarkers, lesion volume (Fig. 4A) exhibited the weakest relation to PDDS. BPF (Fig. 4C), followed by GMF (Fig. 4E), thalamic volume (Fig. 4D), and WMF (Fig. 4F), all out‐performed lesion volume.

### Relations between Gaussian mixture model measures and disability

**Figure 5:**
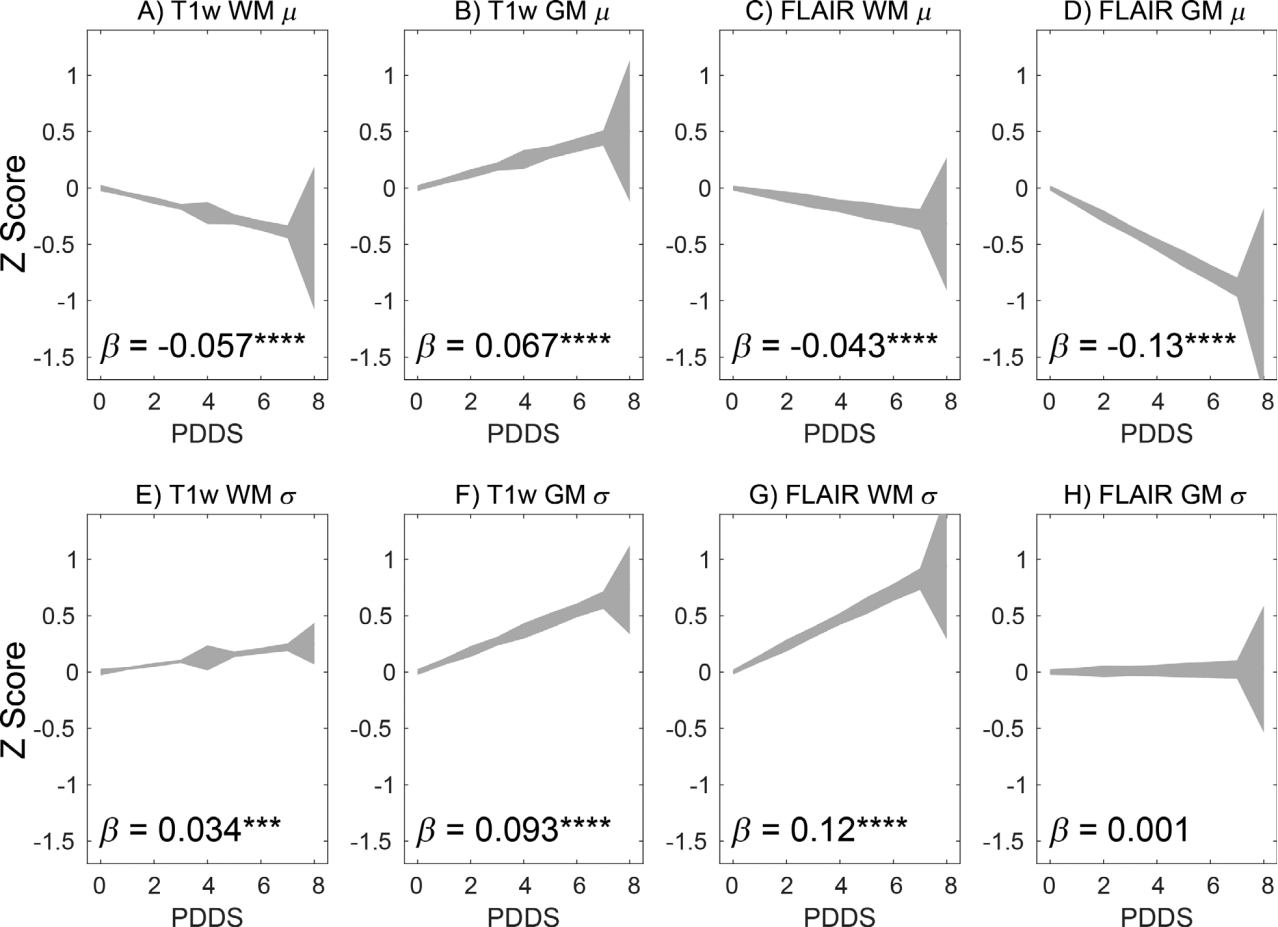
Abnormalities in white matter and gray matter intensity and variability in non‐lesional GM and WM correlate with MS disability. The relationship between imaging measures of MS patients and the patient‐determined disease steps (PDDS) score are shown graphically, along with linear regression *β* values. Imaging measures were *Z* transformed such that different metrics are on the same scale, making *β* values comparable across measures. The panels demonstrate the relationships between PDDS and intensity and variability parameter measures of non‐lesional GM and WM. Asterisks indicate *P* values. *P < 10^−2^; **P < 10^−^^3^; ***P < 10^−^^4^; ****P < 10^−^^5^.

Figure 5 relates GMM measures in GM and WM to PDDS in all MS subtypes (with discrete lesions excluded), displayed as in Figure 4. Several GMM measures exhibited close associations with PDDS. FLAIR GM *μ* (Fig. 5D) and FLAIR WM *σ* (Fig. 5G) yielded regression values (*β* = −0.13 and 0.12, respectively) that were threefold to fourfold greater than the regression value for lesion volume (Fig. 4A) and approximately equivalent to BPF (Fig. 4C). Results describing the relationship between GMM measures and PDDS separated by MS subtype are shown in Table S2 and discussed in Supplemental Results. The relationships between GMM measures and PDDS were predominantly constant across MS subtypes, but when differences emerged, which were small, the relationship was strongest in RRMS. The PDDS corresponds closely to the EDSS but remains a subjective measure. Accordingly, to enhance the reliability and interpretability of the PDDS results, we evaluated these imaging biomarkers in relation to the neuroperformance tests (NPT) within the MSPT and Neuro‐QoL. Concordant results were obtained with the NPT and Neuro‐QoL. The NPT and Neuro‐QoL have three and 12 measures, respectively. Each was reduced to a single composite score using principal component (PC) analysis (individual measures are listed in Table S3). The first PC explained 82% and 60% of the variance of NPT and Neuro‐QoL, respectively. As with PDDS, FLAIR GM *μ* and FLAIR WM *σ* exhibited the strongest relationships with NPT and Neuro‐QoL (Table 3). Moreover, the *t* scores for FLAIR GM *μ* and FLAIR WM *σ* associations with NPT and Neuro‐QoL were approximately five times larger than the *t* score relating WML volume and NPT and Neuro‐QoL. WML‐based measures showed no significant relation to NPT or Neuro‐QoL.

**Table 3:** Neuroperformance and Neuro‐QoL statistical results.

	NPT		Neuro‐QoL	
	*t*	*P*	*t*	*P*
T1w WM *μ*	+7.27	<10^−13^	+0.65	–
T1w GM *μ*	−11.59	<10^−16^	−1.76	–
T1w WM *σ*	−0.11	–	+0.28	–
T1w GM *σ*	−12.26	<10^−16^	+1.01	–
FLAIR WM *μ*	+6.47	<10^−9^	+1.02	–
FLAIR GM *μ*	+26.45	<10^−16^	+3.50	<10^−3^
FLAIR WM *σ*	−26.16	<10^−16^	−4.52	<10^−5^
FLAIR GM *σ*	−4.09	<10^−4^	+0.46	–
WM Les Vol	−5.28	<10^−6^	−1.31	–
WM Les Intens	−8.45	<10^−16^	−1.99	–

Note.

Main effects derived by linear regression of GMM parameters on first principal components of neuroperformance tests (NPT) in the MSPT and Neuro‐QoL scores. *t* values indicate the strength and direction of the relation. Only significant *P* values (uncorrected for multiple comparisons) are listed. The effect size of several GMM parameters was larger than the effect size of lesion burden metrics. Significant effects of *σ* generally were negative, indicating that greater signal variability is related to greater impairment. The direction of the effect of *μ* on performance was not consistent. GMM, Gaussian mixture modeling.

### Accounting for both brain volume and Gaussian mixture model parameters improves correlation with disability

A subset of GMM parameters as well as volume‐based measures was strongly correlated with PDDS. Specifically, this was true of BPF (*β* = −0.16), FLAIR GM *μ* (*β* = −0.13), and FLAIR WM *σ* (*β* = +0.12). To address the question of whether these parameters provide unique versus redundant information, we performed LASSO regression,^2928^ which assigns weights to factors contributing to an outcome measure (i.e., PDDS) under a constraint (formally, L1‐penalized regression) that eliminates weak or, critically, redundant contributions. An important consideration of LASSO regression is the biological interpretability of the resulting model. Inclusion of a variable in the model indicates that, compared to other included variables, a given variable contains additional explanatory power. Exclusion of a variable does not necessarily imply that it lacks predictive power, only that the added predictive power is less than some tuning penalty. Importantly, given two equally predictive and highly correlated variables subject to different sampling error, LASSO will include the variable with less sampling error. The LASSO model was computed using 90% of randomly selected participants and tested in the remaining 10%. Two lesion metrics (Fig. 4A and B), 4 brain volume metrics (Fig. 4C–F), and eight GMM metrics (Fig. 5) were included in the model, which identified two significant predictors of PDDS: BPF (*β* = −0.26) and FLAIR GM *μ* (*β* = −0.12). All other variables were eliminated. This result indicates that brain volume and FLAIR GM *μ* independently contribute to correlations with disability. Applying the optimized model to the 10% held‐out data revealed a significant correlation between predicted and measured PDDS (*R* ^2^ = 0.36, *P* < 10^−16^).

## Discussion

MRIs are performed routinely in MS patients. We hypothesized that, by utilizing T1w and FLAIR intensities, additional impactful information could be obtained from routine clinical MRIs. We developed a technique to standardize T1w and FLAIR intensity histograms derived from images obtained with clinically available MRI sequences. This technique was applied to data from previously acquired datasets from over 5000 MS patients and identifying relationships with clinical disability. GMM of the standardized histograms yielded measures that separated MS patients from healthy controls and distinguished between relapsing MS versus progressive MS. The two GMM measures most correlated with disability measures were GM mean intensity (*μ*) and non‐lesional WM intensity variability (*σ*) in FLAIR images. Since WM *σ* was evaluated outside of discrete lesions, this measure pertains to NAWM. LASSO regression demonstrated that GMM analysis of standardized intensity histograms contributed information relating to disability that was independent of lesion burden and atrophy measures. Specifically, LASSO identified FLAIR GM *μ* which may reflect sensitivity to cortical lesions not readily appreciable on visual inspection of the images. This novel analysis technique provides quantitative insights into non‐lesional WM pathology in MS and its relation to disability. Moreover, this powerful technique provides a practical method that can be used to derive quantitative information from clinical brain images, including those acquired previously such as MRIs done in prior clinical trials.

T1 and T2 are intrinsic physical properties of neural tissue^29^ that depend on cellular composition.^30^, ^31^ Absolute measurement of T1 and T2 can be obtained using multiecho MRI sequences.^12^ Such quantitative measures can serve as biomarkers of disease.^32^ MS‐related changes in T1 and T2 are well documented in both the WM and GM, within^33^ and outside^34^ discrete lesions. For example, T2 is prolonged in MS NAWM.^34^ T1 and T2 are proportionally reflected in T1w and T2w (FLAIR) images in a manner that depends on sequence parameters (i.e., TR, TE, presence of suppression pulses, etc.). Variations in these parameters among clinical scans preclude direct recovery of absolute T1 and T2 from T1w and T2w images. The present histogram normalization technique overcomes this barrier by representing T1w and T2w data in a standard bivariate intensity space. Standardized intensities are informationally equivalent to absolutely measured T1 and T2^19^ assuming that T1w and T2w images encode a linear combination of T1 and T2. In practice, this assumption is only approximate as reconstructed image intensities depend on T1 and T2 nonlinearly.^35^ Nevertheless, we have demonstrated the utility of our approach using T1w and FLAIR imaging data. The fundamentals underlying this approach easily accommodate additional imaging contrasts (e.g., susceptibility‐weighted images).

The present results using our novel analysis method are concordant with results obtained using quantitative relaxometry but without the technical challenges of the latter procedure. Our results recapitulate that WM T2 (FLAIR) heterogeneity is increased in MS compared to controls^36^ and in progressive MS compared to relapsing MS.^37^ Thus, results derived from the present method parallel results obtained with quantitative T1 or T2 imaging,^38^ while being accessible in clinically acquired data standardized imaging data.

The present findings provide several insights. Intensity‐based measures derived from the GMM approach separated RRMS from progressive disease but did not distinguish between progressive disease subtypes (PPMS vs. SPMS vs. PRMS). This result is consistent with the concept that all progressive subtypes have similar underlying tissue damage, likely representing the same underling pathologic process. Second, we compared the GMM intensity‐based method to lesion volume metrics and estimates of atrophy using BPF, WMF, GMF, and thalamic volume, head to head. The present intensity‐based measures and volume‐based measures were independently correlated with MS‐related disability. Hence, consideration of both could enhance tracking of disease dynamics. Third, the variability in FLAIR signal intensity in NAWM was strongly correlated with disability. T2 (FLAIR) correlates with myelin content, axon count and other histological features in post‐mortem MS tissue.^39^ T2 changes are not homogenously distributed across NAWM.^37^ The most abnormal areas are prone to developing lesions in the future.^40^ One possible contributor to this observation could be small, subresolution discrete lesions which manifest as signal variability in NAWM. Speculatively, the present normalized T2 (FLAIR) signal properties may reflect this same histopathological variability evident in quantitative relaxometry and ex vivo imaging. FLAIR signal variability was approximately fourfold more strongly correlated with disability compared to FLAIR mean intensity. This result motivates future study of the pathological basis of this finding.

Focal WMLs remain the imaging hallmark of MS diagnosis.^41^ However, lesion burden is known to be a poor predictor of disability^9^ especially at later stages of the disease.^42^ Models that incorporate lesion location are moderately more predictive.^43^ Pathology outside of discrete lesions is increasingly recognized as important in MS.^44^ Thus, it is noteworthy that the GMM parameter FLAIR WM *σ* (Table 2) exhibited an AUC of 0.84 for discriminating controls versus MS patients. From a statistical perspective, an AUC of 0.84 is significant. However, nonunity is congruent with the observation that NAWM abnormalities are variably present at least partially related to level of disability (Fig. 5). Whereas immune‐mediated inflammation is a critical component of lesion pathology,^45^ abnormalities in NAWM may be more related to injury and repair.^46^ We observed a less strong relationship between GMM parameters and disability in progressive patients compared to relapsing patients, in agreement with prior studies.^47^ This result may reflect different pathologies in relapsing versus progressive MS, and it may also reflect the contributions of spinal cord pathology to disability in progressive MS,^48^ which was not assessed in this study.

The present analysis method provides estimates of GM integrity as well as WM measures. GM damage is increasingly recognized as a driver of MS‐related disability.^49^ In parallel to WM abnormalities, GM can contain discrete lesions, detectable only with specialized sequences, as well as diffuse abnormalities. GM pathology may affect neuron cell bodies, axons, dendrites, myelin, glia, and extracellular matrix.^12^ GM disease has been related to WML burden,^50^ but the causal mechanism underlying this relation remains unclear. Quantitative GM T1w signal abnormalities have been associated with clinical status.^38^ We demonstrated that changes in T1w and FLAIR signal intensity within GM significantly correlate with clinical disability. It is possible that the present technique can quantify subtle changes in T1 and FLAIR associated with GM lesions as well as diffuse abnormalities.

Limitations of the present study include that the analyses were cross‐sectional and drawn from an observational cohort. Longitudinal, prospective analyses are planned to clarify the short‐ and long‐term correlates and predictive power of abnormalities in GMM parameters. The MS PATHS protocol does not include spinal imaging; thus, we could not assess contributions of spinal cord pathology to MS‐related disability. In MS PATHS, clinical subtype is self‐reported, and the PDDS is a subjective patient‐reported outcome. However, the MSPT was included in the present work and provided objective support for the results.

These analyses used a standard MRI protocol at 3T. Theoretically, this approach can be applied to other datasets obtained at lower field strengths or nonstandardized imaging acquisition. If confirmed, it may be possible to use this technique to analyze archival clinical trial data and other large, pre‐existing databases. This method might be particularly promising in the reanalysis of prior trials of progressive MS in which results were equivocal or only benefited subgroups of patients. The present intensity normalization technique does have some limitations. First, it relies on the alignment of a measured histogram to a reference which can be affected by subtle movement impacting numeric results in subtle ways. In the present work, there were no strong correlations between alignment error and outcomes of interest (see Supplemental Methods). Additionally, while intensity alignment transforms images to a standard intensity space, the numerical values remain arbitrary precluding direct comparison to prior quantitative relaxometry results. Since this study uses standardized imaging protocols, comprehensive evaluation of interprotocol analysis is reserved for future work.

## Conclusions

We report a novel technique for quantification of non‐lesional abnormalities in WM and GM in MS using data obtained with clinically available MR sequences. Using this new approach, we demonstrate diffuse changes in GM and WM in MS patients compared to healthy controls and differences between relapsing versus progressive disease. Importantly, these changes were more strongly correlated with disability than conventional lesion‐based metrics and comparably correlated with volume‐based metrics, reinforcing that non‐lesional abnormalities are a critical component of MS pathophysiology. Our results also support the notion that progressive non‐lesional pathology is similar across progressive subtypes. The present approach enables quantification of abnormalities using conventional MRI protocols, allowing this technique to be used in clinical populations. Future work will investigate the power of this approach to predict disease progression and monitor response to therapy.

## Author Contributions

MRB and AZS conceived of the study, developed the analytical tools, analyzed the data, and drafted the manuscript. AT and JS developed analytical tools and analyzed the data. RAR, EF, and SJ conceived of the study, oversaw the acquisition and distribution of the data, and assisted in interpreting the data. AHC, TLSB, and RTN oversaw the study, interpreted the data, and drafted the manuscript. All authors revised the final manuscript.

## Conflict of Interest

MS PATHS is funded by Biogen. MRB, AZS, AT, and JS have no disclosures. RAR and EF are employees of Biogen, Inc. SJ received honoraria from Siemens, RadNet and St Jude Children's Research Hospital as well as research support from Biogen, St Jude, National Institutes of Health and performed consultation services for Monteris and Eisai. AHC was supported by the Manny & Rosalyn Rosenthal – Dr. John L. Trotter MS Center Chair in Neuroimmunology and has consulted for or received honoraria from Biogen, Celgene, EMD Serono/Merck, Genentech, Novartis, Greenwich Biosciences, Janssen Pharmaceuticals, and Roche, and is a site principal investigator in contracted research from Genentech and EMD Serono. TLSB is funded by the NIH, Alzheimer's Association, Barnes‐Jewish Hospital Foundation and Avid Radiopharmaceuticals, is a site investigator in clinical trials sponsored by Avid Radiopharmaceutical, Eli Lilly, Biogen, Janssen and Roche, and is on the Speaker's Bureau for Biogen. RTN has consulted for Alexion, Biogen, EMD Serono, Celgene, Genentech, Genzyme, NervGen Pharma, Novartis, Third Rock Ventures, Viela Bio. The above disclosures do not have direct bearing on this work, and the authors report no other conflicts of interest.

## Supporting information


**Supplemental Methods.** Detailed accounting and algebraic description of analysis methods summarized in the main text.
**Supplemental Results.** Complementary results to the main text that perform PDDS ~ GMM analyses by each MS subtype.
**Table S1.** Numerical statistical results corresponding to results shown in Figure 3.
**Table S2.** Statistical results corresponding to the results discussed in Supplemental Results.
**Table S3.** Correlation analysis between GMM and lesion parameters with individual components of the MSPT and Neuro‐QoL.
**Figure S1.** Histogram of single subject alignment error, a quality control measure.
**Figure S2.** Example images following intensity normalization.
**Figure S3.** Correspondence between two lesion segmentation strategies.
