# Supplementary material for: Quantitative signal properties from standardized MRIs correlate with multiple sclerosis disability
Source: Ann Clin Transl Neurol. 2021 May 4;8(5):1096–109. doi: 10.1002/acn3.51354 (PMC8108425; doi:10.1002/acn3.51354)
Supplement: Supplementary file 1 — Supplemental Methods. Detailed accounting and algebraic description of analysis methods summarized in the main text. Supplemental Results. Complementary results to the main text that perform PDDS ~ GMM analyses by each MS subtype. Table S1. Numerical statistical results corresponding to results shown in Figure 3. Table S2. Statistical results corresponding to the results discussed in Supplemental Results. Table S3. Correlation analysis between GMM and lesion parameters with individual components of the MSPT and Neuro‐QoL. Figure S1. Histogram of single subject alignment error, a quality control measure. Figure S2. Example images following intensity normalization. Figure S3. Correspondence between two lesion segmentation strategies. [file ACN3-8-1096-s001.docx]

**SUPPLEMENTAL MATERIAL**

**Supplemental Methods.**

*Imaging Parameters*

Imaging data consisted of standardized 3D T1 and FLAIR image acquired on Siemens scanners. The T1w MPRAGE image had a field of view of 256mm, a TR of 2.3 s, a TE of 2.98 ms, TI of 900 ms, and a resolution of 1mm isotropic voxels. The T2w FLAIR image had a field of view of 256mm, a TR of 5 s, a TE of 393 ms, TI of 1800 ms, and a resolution of 1mm isotropic voxels. These parameters were used for all images acquired and included in the present study. Each site used their standard head coil.

*Image Pre-processing*

Imaging data consisted of 1mm^3^ isotropic voxel, T1-weighted (T1w) MP-RAGE and 3D-FLAIR sequences with identical fields of view. The T1w and FLAIR images underwent brain extraction and bias field correction using the BET and FAST modules in the FSL toolbox ^27^. The FLAIR and T1w images were affine coregistered (rigid body + cardinal axis scaling, typically on the order of 0.1%; 6+3=9 parameters total) using in-house software (http://4dfp.readthedocs.io). Voxelwise intensity values in the brain-masked (BET) and spatially coregistered images were normalized to achieve a mode value of 1,000 (multiplying all voxel values by a constant) to accelerate subsequent intensity normalization.

*Univariate and Bivariate Image Intensity Histogram Calculation*

The fundamental principle underlying the present analyses is standardization of bivariate intensity (T1w$\times$ FLAIR) histogram shape to match normative data. Computational steps of the analyses involve statistical techniques applied to univariate and bivariate intensity histograms. Bivariate histograms were created representing T1w/FLAIR voxel intensities on the horizontal/vertical axes, respectively. Histogram peaks occurred at specific loci in intensity space corresponding to distinct tissue classes (GM, WM, CSF; Figure 1). Individual participant histograms exhibited grossly similar shapes in healthy controls and MS patients but subtly varied in scale and skew across individuals (dashed lines in Figure 1), most likely owing to a combination of disease-relevant processes and disease-irrelevant differences such as scanner calibration and differences in head coils. The following procedure enabled standardization of bivariate histograms, thereby facilitating precise tissue classification and comparison.

*Image Intensity Histogram Normalization*

Image intensity histogram normalization was accomplished in two steps. The affine transformation representing the operation which aligns the single participant bivariate histogram image to the target bivariate histogram image is calculated. This affine transformation then is applied voxelwise to the bispectral intensity histogram. (This same technique can be generalized to multispectral image data.)

Affine transformation combines two operations: linear mapping and translation. Let $A$ be the affine transform that aligns the single participant bivariate histogram to the target bivariate histogram. This has the form

$$A=\left[ \begin{matrix} {sc}_{1} & {sh}_{1} & {tr}_{1} \\ {sh}_{2} & {sc}_{2} & {tr}_{2} \\ 0 & 0 & 1 \end{matrix} \right]$$

where *sc*, *sh*, and *tr* refer to scaling, shearing and translation in either the first or second image histogram dimension. Determination of $A$ uses regular step gradient descent (van der Bom *et al.*, 2011) to minimize an objective function between the affine transformed single participant bivariate histogram and the target. The objective function in this case is the mean squared error between the aligned image and the target.

The affine transform is next applied voxelwise to the intensity in the bispectral image data. Let $V_{k}=\left[ \begin{matrix} v_{k,T1} & v_{k,FLAIR} & 1 \end{matrix} \right]^{T}$ where $v$ is the voxelwise intensity at voxel $k$. The normalized intensity values are thus defined

$$\tilde{V}_{k}=A{\cdot V}_{k}$$

This transformation is then applied across all voxel to create intensity normalized images.

*Reference Histogram Calculation*

A reference histogram (target) was created by iterative analysis of data obtained in 100 control participants. At each iteration, the bivariate intensity histogram of each control participant was affine registered (6 parameters) to the current target using the above described technique. The average of the resulting aligned bivariate histograms then served as the target for subsequent iterations. The iterative procedure was initialized by assigning one randomly selected participant as target. In the present data, convergence (no change in affine transform parameters to 2 decimal places) was achieved after 5 iterations.

*Single Participant Histogram Intensity Normalization*

Intensity normalization of all participants (n=5,030) was achieved by affine registration of individual bivariate histograms to the reference histogram using the same algorithm detailed above. Alignment error in each individual was compared to a fixed criterion determined by visual inspection of a representative sample (Supplemental Figure 1). Individuals with histogram alignment error above this threshold (n=28, 0.6%) were excluded from further analyses. Unsatisfactory histogram alignment was most often attributable to head motion or image artifact, e.g., from dental implants. No strong correlation was observed between alignment error and quantities of interest reported below (all r<0.1). Figure 1 illustrates bivariate histograms in two example participants (1 control, 1 MS patient) before and after intensity normalization. The normalization parameters obtained in each participant were applied voxelwise to the T1w and FLAIR data, thereby generating images whose bivariate histograms closely approximated the normative reference. The normalized data then were split into two univariate histograms representing normalized T1w and FLAIR image intensity distributions. These univariate, normalized histograms were subsequently subjected to comparisons of interest, e.g., MS patients vs. controls.

*Tissue and Lesion Segmentation*

Intensity normalization generated T1w and FLAIR data in which tissue classes (CSF, WM, GM, lesion) were represented by as narrow distributions about centroids in a bivariate intensity space. Tissue segmentation was achieved by implementing a procedure similar to k-means clustering to compute voxelwise tissue class assignments. Tissue class centroids were defined *a priori* by manual segmentation of representative images. Each voxel was assigned a continuous score between 0 and 1 representing membership in each tissue class. The algebra of this technique is detailed below.

| Symbol | Definition |
| --- | --- |
| $i$ | index of tissue class |
| $j$ | index of contrast in multispectral image data (e.g., T1w vs. FLAIR) |
| $r_{j}$ | useful range of image values for contrast $j$ |
| $\bar{f}_{ij}$ | coordinate of tissue class centroid in contrast space |
| $f_{j}$ | coordinate of image voxel values in contrast space |
| $d_{i}^{2}$ | standardized squared distance (in contrast space) of voxel from tissue class centroid |
| $u_{i}$ | normalized class membership |

For any multispectral image voxel, the standardized squared distance from the $i$^th^ tissue class centroid was

$d_{i}^{2}=\sum_{j} \left[ \left( f_{j}-\bar{f}_{ij} \right)/{r_{j}} \right]^{2}$,

where standardization means taking into account the useful intensity range, $r_{j}$. For any multispectral voxel, define raw membership in class $i$ as $1/{d_{i}^{2}}$ and the normalized membership in class $i$ as

$u_{i}=\left( 1/{d_{i}^{2}} \right)/{\sum_{k} \left( 1/{d_{k}^{2}} \right)}$,

where $k$ is a dummy index of summation. Thus, $\sum_{i} u_{i}=1$. In the following, all references to class membership assume normalization. If the tissue class centroids are fixed in multispectral intensity space, then the preceding definitions are sufficient to assign tissue class membership to each multispectral image voxel.

To define tissue class boundaries in image space, we began by computing class membership gradients with respect to image values. For notational convenience, define $D_{j}\equiv\partial/{\partial f_{j}}$. Then

$D_{j}d_{i}^{2}=2\sum_{j} \left[ \left( f_{j}-\bar{f}_{ij} \right)/{r_{j}^{2}} \right]$,

$D_{j}\left( 1/{d_{i}^{2}} \right)=-{(d_{i}^{2})}^{-2}D_{j}d_{i}^{2}$,

$D_{j}u_{i}=\left[ D_{j}\left( 1/{d_{i}^{2}} \right)-\left( 1/{d_{i}^{2}} \right){(D}_{j}g)/g \right]/{(g\cdot r_{j}})$,

where $g=\sum_{k} \left( 1/{d_{k}^{2}} \right)$ and $D_{j}g=D_{j}\sum_{k} \left( 1/{d_{k}^{2}} \right)$.

We wish to compute tissue class membership gradients in image space, $\nabla u_{i}$. This was done by application of the chain rule. Thus,

$\nabla u_{i}=\left[ \frac{\partial u_{i}}{\partial f_{j}}\frac{\partial f_{j}}{\partial x} \right]\hat{i}+\left[ \frac{\partial u_{i}}{\partial f_{j}}\frac{\partial f_{j}}{\partial y} \right]\hat{j}+\left[ \frac{\partial u_{i}}{\partial f_{j}}\frac{\partial f_{j}}{\partial z} \right]\hat{k}$,

where $\left\{ \hat{i,} \hat{j}, \hat{k} \right\}$ are cardinal ($x, y, z)$ axis unit vectors in image space.

Boundaries between tissue compartments correspond to loci at which two class memberships were dominant and approximately equal. In the archetypical case, $u_{1}=u_{2}=0.5$, taking classes 1 and 2 as examples. More generally, the tissue class boundary corresponds to $u_{1}\approx u_{2}$, although $u_{1}+u_{2}<1$, because of finite representation of other tissue classes ($\sum_{i} u_{i}=1$). Imagine that the tissue class boundary normal points exactly in the $x$-direction. Then, the boundary may be found where $\left| u_{2}({du_{1}}/{dx})-u_{1}({du_{2}}/{dx}) \right|$ was maximal. In 3-D image space, this expression generalized to the locus where $\left| u_{2}\nabla u_{1}-u_{1}\nabla u_{2} \right|$ was maximal. A rationale for this expression may be seen in the equality,

$u_{2}\nabla u_{1}-u_{1}\nabla u_{2}=(u_{1}^{2}+u_{2}^{2})\nabla\tan^{-1} ({u_{1}}/{u_{2}})$.

In preliminary analyses, it was observed that healthy controls often exhibited non-zero, occasionally large, voxelwise lesion scores that did not correspond to MS lesions. Non-zero lesion scores in controls occurred in WM abnormalities attributable to small vessel disease and at tissue class boundaries (e.g., choroid plexus, bone interfaces) owing to partial volume effects. These non-MS lesion tissues occur in a stereotypical topography. To minimize false detection of MS lesions, we implemented a correction procedure that down-weighted lesion scores according to the mean lesion score image in the controls. Accordingly, the single participant lesion score image was Z-scored with respect to the control distribution on a voxelwise basis, $\tilde{u}_{l}^{s,x}=\left( u_{l}^{s,x}-\left\langle u_{l}^{x} \right\rangle_{s\in C} \right)/{\sigma\left( u_{l}^{x} \right)_{s\in C}}$ where $u_{l}^{s,x}$ is the lesion score for participant $s$ at voxel $x$ and $\left\langle\cdot\right\rangle_{s\in C}$ and $\sigma\left( \cdot\right)_{s\in C}$ represented the mean and standard deviation across participants in the control group, respectively. By applying this transform, MS lesions are isolated and non-MS lesions or lesion-appearing tissue is excluded. Voxels were classified as “lesion” if their corrected lesion score exceeded a threshold designed to minimize false negative assignment. This choice ensured that MS lesions were reliably excluded from NAWM and GM at the cost of also excluding borderline voxels. Thus, normalized intensity-based lesion segmentation generated a lesion count and total lesion volume in each individual.

*Summary of Lesion Segmentation Procedure*

For a randomly selected patient, Supplemental Figure 2 shows the raw T1 and FLAIR images and completely processed images. Aside from the removal of extra-axial structures, the most salient difference in these images is the change of scale which facilitates comparison across individuals. The lesion score is also shown. Detailed inspection of this image reveals that WMLs are well captured. However, there are a number of apparently “false-positive” voxels. These are not suppressed because the exclusion of all WMLs is critical to the scientific inference of this study. Therefore, a small number or excluded non-lesion voxels is preferable to the erroneous inclusion of lesion voxels. By way of comparison, the segmentation result of an alternate algorithm (MSPie, described in main text) is provided. Visual inspection reveals close concordance between these two results.

*Gaussian Mixture Modeling and Statistical Analyses*

Contrasts of interest, e.g., differences between healthy control participants vs. patients with MS, were computed by analysis of normalized, univariate T1w and FLAIR intensity distributions within tissue classes determined by Gaussian mixture modeling (GMM). GMM expresses an arbitrary distribution, $Y$, as the sum of normal Gaussian components:

$Y=\sum_{k=1}^{n} a_{k}N\left( \mu_{k},\sigma_{k}^{2} \right)$,

where $N$ denotes a normal distribution, $n$ is the number of components, and $a_{k}$ are scalar weights. The key parameters characterizing each component (indexed by $k$) are $\mu_{k}$ (mean) and $\sigma_{k}^{2}$ (variance). We selected the number of components, $n$, using the Akaike information criterion (AIC), independently for the T1w and FLAIR intensity distributions. Following determination of $n$, the other parameters were fit using an expectation maximization algorithm ^28^. To facilitate computational speed, the individual Gaussians were initialized by setting $\mu_{k}$ to tissue class centroids as defined above. By systematic evaluation of the AIC over $n=\left[ 1,2,3,4,5 \right]$, we determined that optimal histogram fits were obtained at $n=3$ for T1w (*t*=25.4, p<10^-32^), and $n=4$ for FLAIR (*t*=115.7, p<10^-32^). Thus, GMM components in normalized T1w histograms corresponded to CSF, GM, and WM; GMM component normalized FLAIR histograms corresponded to CSF, WM, GM, and focal hyperintensities (lesions; Figure 2A,B). The component means ($\mu$) and standard deviations ($\sigma$) corresponding to GM and WM in the T1w and FLAIR data were carried forward as primary measures of interest. GMM fitting was carried out twice, once with lesions included and again with lesions excluded via masking. Statistical significance was assessed by fitting linear models with MS diagnosis, MS subtype, and disability metrics as factors, after adjusting for age and gender.

*Quality Assurance*

Quality assurance was achieved in three stages. At the time of acquisition, images are visually inspected by technicians for adequate quality. When received at Biogen, images are processed through an automatic QA/QC ingest script and flagged for visual review if incomplete or not acquired with the right sequence parameters. Images are then analyzed by an automated image analysis program called MS PIE (described below) which, following brain and lesion segmentation, automatically assesses quality and flags cases for visual review. During the registration of the T1 and FLAIR image, artifacts (e.g., movement) which preclude good alignment are detected via an objective function which measures goodness of alignment. Finally, images which fail intensity normalization are identified using an automated procedure. In this way, only images relatively unconfounded by artifact are included in the analysis.

**Supplemental Results**

*Lesion Masks Derived from the Present Lesion Score Approach and MSPie are Similar*

This report presents two different lesion segmentation approaches for comparison. MSPie is automated and provides a binary lesion segmentation. Lesion scores are continuous variables allowing for lesion segmentation with varying sensitivities and specificities. To determine if similar results can be recovered from both, we performed an ROC analysis with the MSPie result serving as the label and the lesion score result serving as the variable of interest. Supplemental Figure 3 shows that, across subjects, there is good agreement between these two approaches.

*Relationship of GMM Parameters with Disability among Different MS Subtypes*

In the main text, we report the association of PDDS and GMM parameters combining all MS subtypes. Here we break down this relationship by MS subtype. ANCOVA was used to evaluate associations between GMM parameters and PDDS with MS subtype as factor. Three inferences were derived: main effect of MS subtype, main effect of PDDS, interaction between MS subtype and PDDS (Supplementary Table 1A). For FLAIR GM μ, T1 GM σ, and FLAIR WM σ associations with PDDS were largest in the RRMS group as indicated by the large magnitude β coefficients (Supplementary Table 1B). This result suggests a closer relationship between GMM parameters and disability in the RRMS compared to progressive MS.

**Supplemental Table 1.** Effect of group (Control, MS subtype) on GMM parameters

| **Lesion Included** | | | | |
| --- | --- | --- | --- | --- |
|  | μ | | σ | |
|  | F_4,5006_ | p | F_4,5006_ | p |
| T1w WM | 12.96 | <10^-9^ | 0.45 | 0.78 |
| T1w GM | 12.81 | <10^-9^ | 16.26 | <10^-12^ |
| FLAIR WM | 9.55 | <10^-6^ | 55.52 | <10^-45^ |
| FLAIR GM | 46.16 | <10^-37^ | 6.46 | <10^-4^ |
| **Lesion Excluded** | | | | |
|  | μ | | σ | |
|  | F_4,5006_ | p | F_4,5006_ | p |
| T1w WM | 8.00 | <10^-5^ | 0.63 | 0.64 |
| T1w GM | 16.22 | <10^-12^ | 20.72 | <10^-16^ |
| FLAIR WM | 9.62 | <10^-7^ | 55.73 | <10^-45^ |
| FLAIR GM | 65.89 | <10^-53^ | 0.48 | 0.75 |

GMM parameters were submitted to ANOVA with groups (Controls, RRMS, SPMS, PPMS, PRMS) as factor. This table presents F values and p values related to the main effect of group membership for the GMM μ and σ parameters. p values are uncorrected. Post-hoc comparisons between MS subtypes are shown in Figure 3. The most salient points in this analysis is that the principal findings are not significantly modulated by the exclusion of lesions demonstrating that these effects are driven by non-lesional differences. The most significant findings are characterized by very large F values.

**Supplemental Table 2.** Group-wise Statistical Tests: MS Subtypes by Disability Interaction

| A) | μ | | | | | | | | | σ | | | | | | | |
| --- | --- | --- | --- | --- | --- | --- | --- | --- | --- | --- | --- | --- | --- | --- | --- | --- | --- |
|  | MS Subtype | | | PDDS | | | Interaction | | | MS Subtype | | | PDDS | | | Interaction | |
|  | F | p | | F | p | | F | p | | F | p | | F | p | | F | p |
| T1 WM | 0.23 | 0.87 | | 35.75 | <10^-8^ | | 0.67 | 0.57 | | 0.09 | 0.97 | | 0.49 | 0.48 | | 0.18 | 0.91 |
| T1 GM | 0.23 | 0.88 | | 43.56 | <10^-10^ | | 0.89 | 0.45 | | 3.51 | 0.015 | | 59.03 | <10^-13^ | | 3.40 | 0.017 |
| FLAIR WM | 2.11 | 0.097 | | 10.28 | 0.0014 | | 1.12 | 0.34 | | 6.51 | 0.0002 | | 116.19 | <10^-26^ | | 2.78 | 0.040 |
| FLAIR GM | 3.64 | 0.012 | | 159.80 | <10^-35^ | | 4.45 | 0.004 | | 1.21 | 0.30 | | 0.62 | 0.42 | | 1.27 | 0.28 |
| B) | | | RRMS | | | SPMS | | | PPMS | | | PRMS | | |  |  |  |
| T1 GM σ | | | 0.53  [0.42, 0.64] | | | 0.26  [0.10, 0.41] | | | 0.21  [0.01, 0.41] | | | 0.49  [0.31, 0.68] | | |  |  |  |
| FLAIR GM μ | | | -0.87  [-0.99, -0.75] | | | -0.53  [-0.71, -0.34] | | | -0.69  [-0.98, -0.40] | | | -0.52  [-0.77, -0.26] | | |  |  |  |
| FLAIR WM σ | | | 0.84  [0.71, 0.97] | | | 0.65  [0.43, 0.87] | | | 0.43  [0.12, 0.74] | | | 0.58  [0.29, 0.87] | | |  |  |  |

A) GMM parameters were subjected to ANCOVA with MS subtype (RRMS, SPMS, PPMS, PRMS) and disability (PDDS) as factors. Importantly, the interaction between MS subtype and disability was explicitly modeled. F values and p values are shown. p values are uncorrected. B) GMM parameters were subjected to a model with MS subtype and PDDS as main factors with the interaction additionally modeled. Linear contrasts were extracted from models with significant (p<0.05) interaction terms suggesting a differential relationship between GMM parameter and PDDS based on MS subtype (Supplemental Table 1). Regression β coefficients relating GMM parameters with PDDS in individual MS subtypes were calculated. 95% CI are shown in square brackets. β coefficients are a useful test statistic because they encode the strength and direction of the effect.

**Supplemental Table 3.** Correlations between GMM parameters and individual items in the neuroperformance tests and Neuro-QoL.

Correlation of GMM parameters and lesion volume metrics with individual components of the neuroperformance tests and Neuro-QoL. Correlation coefficients are Pearson coefficients. Color intensity encodes magnitude of relationship and color indicates sign. Note that correlations of greater magnitude were observed for GMM parameters of non-lesional GM and WM versus with lesion volume and intensity measures. T1 = T1w image, FL = FLAIR, WM = White Matter, GM = Grey Matter.

**Supplemental Figure 1**


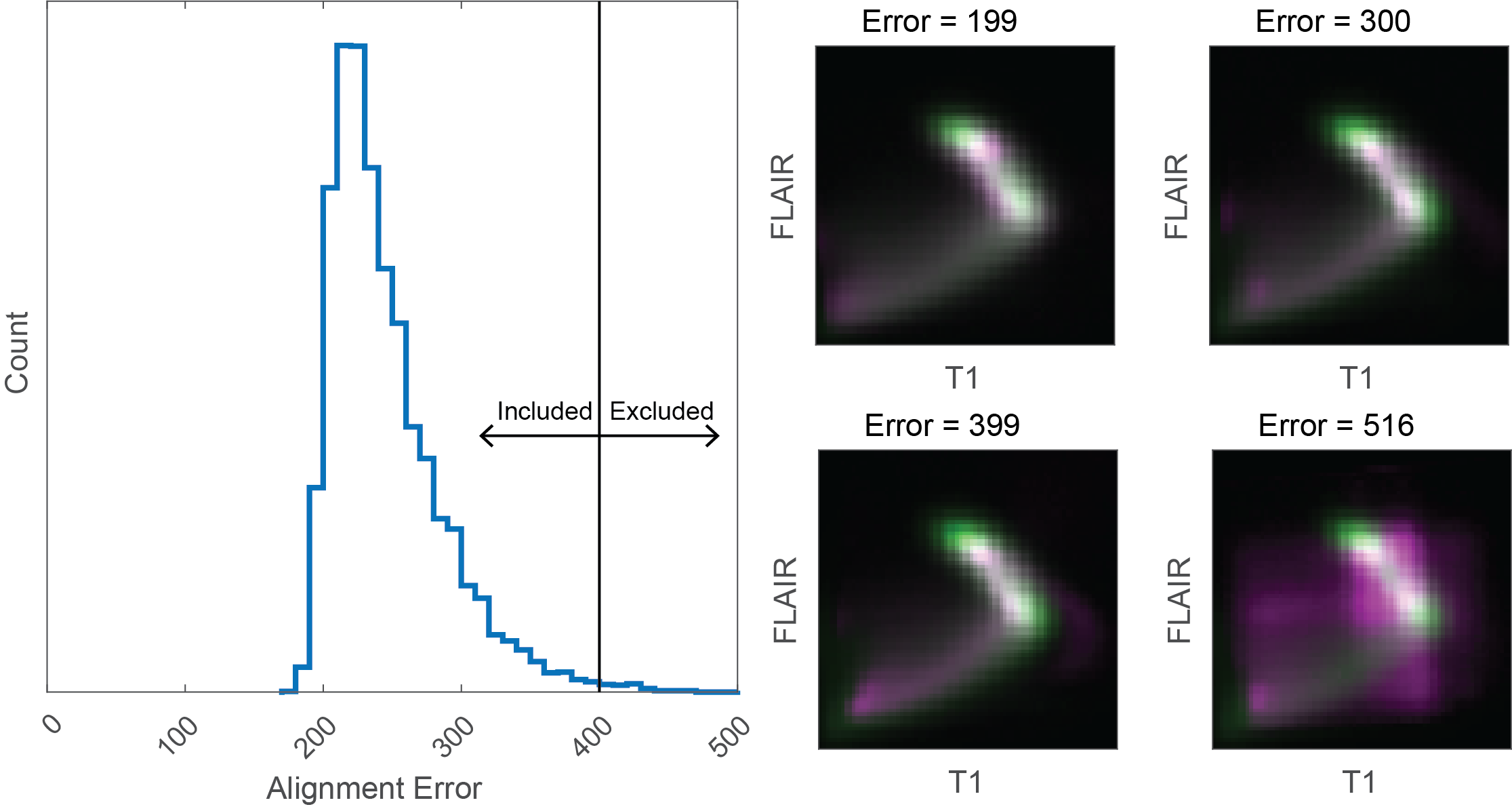


Supplemental Figure 1. **Single Participant Alignment Error.** Left panel shows a histogram of the alignment error, calculated by the basis function, across all MS patients included in the study. Most MS patients have errors between 200 and 300 with a long right-handed tail. Single participant alignments corresponding to error values of approximately 200, 300, 400 and 500 were selected at random. In the right hand panels, the overlap between the intensity normalized bivariate histograms for a single patient (purple) and the target histogram (green) are shown. White shows the overlap. Alignments appear reasonable for errors <= 400.

**Supplemental Figure 2**

**
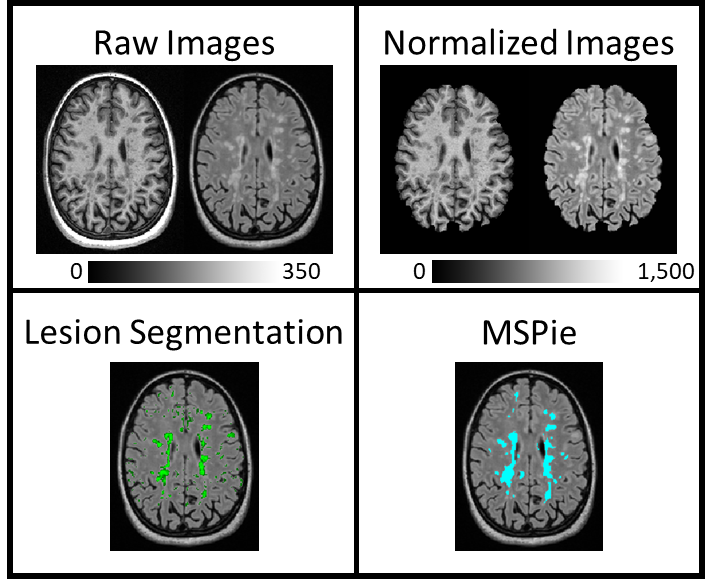
**

Supplemental Figure 2. **Summary of Image Intensity Normalization and Lesion Segmentation Procedure.** Top left quadrant shows the original T1 and FLAIR images with only rigid body spatial registration performed. The top right quadrant shows these same images after all the processing described in this report. Critically, these images remain visually unchanged but the intensity scale is now changed following intensity normalization. The resulting lesion segmentation is shown in the bottom right quadrant. Critically, all major lesions are identified. However, there are a number of false positive voxels. As described in the supplemental text, for the present analysis it is preferable to exclude a small number of non-lesion voxels than to include lesion voxels erroneously. By way of comparison, an alternate lesion segmentation result is shown in the lower right quadrant. Overwhelmingly, these provide converging estimates of lesion burden.

**Supplemental Figure 3**


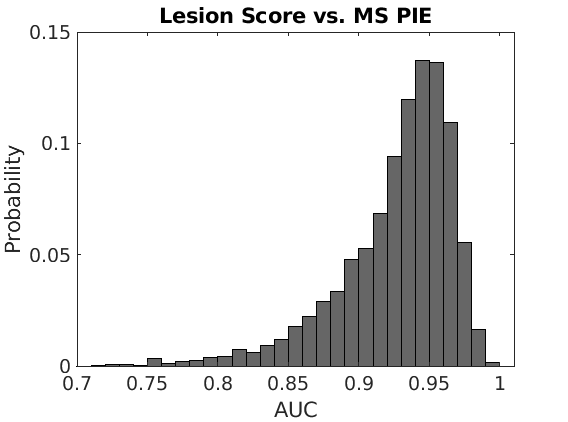


Supplemental Figure 3: **Lesion Topographies Based on Lesion Scores and MSPie are Similar.** For each included subject, we performed an ROC analysis using the MSPie lesion segmentation as label and the whole brain voxelwise lesion score (continuous) as variable of interest. We then tabulated the individual AUC from each ROC analysis and represented those data as a histogram. The key feature of this distribution is that the mean AUC approaches 1 with a tail towards 0.7. This result indicates good agreement between the two lesion segmentation techniques.
